# Evaluation of a Violence-Prevention Programme with Jamaican Primary School Teachers: A Cluster Randomised Trial

**DOI:** 10.3390/ijerph16152797

**Published:** 2019-08-06

**Authors:** Helen Baker-Henningham, Yakeisha Scott, Marsha Bowers, Taja Francis

**Affiliations:** 1 School of Psychology, Bangor University, Bangor LL57 2AS, UK; 2 Caribbean Institute for Health Research, University of the West Indies, Kingston 7, Jamaica

**Keywords:** violence, teacher training, child behaviour, corporal punishment, low- and middle-income country, primary school

## Abstract

This study investigated the effect of a school-based violence prevention programme implemented in Grade 1 classrooms in Jamaican primary schools. Fourteen primary schools were randomly assigned to receive training in classroom behaviour management (*n* = 7 schools, 27 teachers/classrooms) or to a control group (*n* = 7 schools, 28 teachers/classrooms). Four children from each class were randomly selected to participate in the evaluation (*n* = 220 children). Teachers were trained through a combination of workshop and in-class support sessions, and received a mean of 11.5 h of training (range = 3–20) over 8 months. The primary outcomes were observations of (1) teachers’ use of violence against children and (2) class-wide child aggression. Teachers in intervention schools used significantly less violence against children (effect size (ES) = −0.73); benefits to class-wide child aggression were not significant (ES = −0.20). Intervention teachers also provided a more emotionally supportive classroom environment (ES = 1.22). No benefits were found to class-wide prosocial behaviour, teacher wellbeing, or child mental health. The intervention benefited children’s early learning skills, especially oral language and self-regulation skills (ES = 0.25), although no benefits were found to achievement in maths calculation, reading and spelling. A relatively brief teacher-training programme reduced violence against children by teachers and increased the quality of the classroom environment.

## 1. Introduction

Estimates of past-year prevalence of violence against children indicate that more than three quarters of the world’s children (1.5 billion children) have experienced violence, showing an urgent need for evidence-based interventions to prevent violence against children to be adopted and scaled at the global level [1]. Children are exposed to violence at home, at school and in the community. At school, children are faced with violence from teachers and from peers. The use of corporal punishment at school is prohibited in 131 countries, but there are no legal bans in 68 countries [2]. However, even in countries with a legal ban, corporal punishment is widely used and there is a need for additional programmes to prevent the use of violence against children by teachers [2]. School-based violence prevention programmes are thus an important component of the primary prevention of violence, and can prevent: (i) violence against children by teachers, (ii) aggression among children, and (iii) the early development of antisocial behaviour. There have been few such studies from low- and middle-income countries (LMIC). In a meta-analysis of psychosocial intervention to reduce children’s disruptive behaviour in LMIC, only 3 of the 28 interventions included a teacher-training component; the others involved parent training and/or child training programmes [3]. Furthermore, while there have been a growing number of studies to evaluate programmes aimed at reducing violence against children by parents [4,5,6], there have been few rigorous studies evaluating approaches to reduce violence by teachers [7].

Schools are a logical setting in which to implement preventative interventions, given the facts that children spend a large proportion of their time at school and that they cater to the entire population of children, thus providing the opportunity for universal reach. Exposure to violence has long-term negative effects on children’s development. For example, physical and verbal abuse by caregivers is associated with multiple negative outcomes including increased aggression, poor mental health, delinquent and antisocial behaviour, drug use, poor sexual health and an increased risk for perpetrating child or spousal abuse in adulthood [8,9]. Violence against children by teachers is also associated with poor academic achievement [10,11]. Exposure to high levels of peer aggression at school predict later behavioural difficulties, especially for boys and for children at high risk for developing antisocial behaviour [12]. Early childhood is a particularly sensitive period and experiences in early childhood have long term effects on brain function, cognition and psychosocial functioning, and thus predict physical and mental health over the long term [13,14]. Interventions at school entry, when children are experiencing formal schooling for the first time, thus have an important role in helping to place children on a positive trajectory.

In Jamaica, there is no legal ban against the use of corporal punishment in primary schools and violence against children, including physical and psychological aggression, is common. For example, 92.5% of 1300 children in Grade 5 of primary school reported being physically punished at school during the school year [10]. Child externalizing behavioural difficulties are also common. In a survey of Jamaican 5–6 year old children, the reported prevalence of externalising disorders was 12% [15], and in an efficacy trial, we found that 21% of children attending inner-city community preschools had four or more symptoms of conduct problems. The three in each class with highest levels of conduct problems were observed to display a median of 12 aggressive/destructive acts per hour [16], indicating that inner-city preschool classrooms have significant numbers of children with behavioural problems and are characterised by high levels of aggression. We have conducted several previous studies in Jamaica involving training preschool teachers in classroom behaviour management and how to promote young children’s social and emotional competence (16–18). We found benefits from this teacher-training intervention to the behaviour of high risk children at school and at home [16], class-wide child behaviour [17,18], and teachers’ behaviour management practices [17,18]. Through implementing these studies, and conducting quantitative, qualitative and ongoing process evaluations, we developed the IRIE Classroom Toolbox, a school-based teacher training programme for use with children aged 3–6 years [19]. For this study, we adapted the IRIE Classroom Toolbox to be suitable for use with teachers in the early grades of primary school.

The main aim of this study was to evaluate whether training Grade 1 primary school teachers in classroom behaviour management and how to promote children’s social and emotional competence would reduce teachers’ use of violence against children and the level of class-wide child aggression. We also investigated the effect of the intervention on additional outcomes at the level of the teacher (professional wellbeing), the classroom atmosphere (level of emotional support and class-wide prosocial behaviour) and the individual child (child behavioural difficulties, prosocial behaviour and academic achievement), and conducted in-depth individual interviews with teachers who participated in the training programme to explore their perceptions of the use of harsh punishment as a discipline strategy.

## 2. Materials and Methods

### 2.1. Study Design and Sample

**Figure 1:**
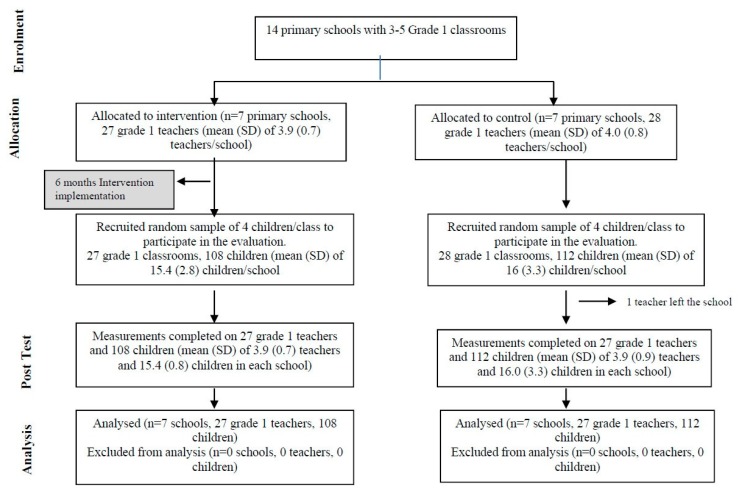
Trial profile.

Fourteen primary schools situated in inner-city areas of Kingston, Jamaica participated in the study (see Figure 1 for the trial profile). Primary schools cater to children aged 6–12 years from Grade 1 to Grade 6. All primary schools that participated in this study had same-age classrooms and each grade was led by a grade co-ordinator who also was a classroom teacher. Primary school was the unit of randomisation to prevent contamination between teachers. Inclusion criteria for schools were (1) had 3–5 Grade 1 classrooms, (2) situated in a specified geographical area, and (3) the principal and all Grade 1 teachers consented to participate in the trial. Exclusion criteria were: (1) attached to a teacher-training college, (2) had competitive entry, and (3) teachers had participated in at least three rounds of a previous study [20]. A list of all schools in the region was prepared and all schools with four Grade 1 classrooms (seven schools) and five Grade 1 classrooms (three schools) meeting the inclusion criteria were selected. A random sample of three schools was then selected from the schools with three Grade 1 classrooms. All teachers of children in Grade 1 classrooms participated in the study.

All children within the Grade 1 classrooms in intervention schools received the intervention. To evaluate the effect of the teacher-training on individual child outcomes, a random sample of four children from each Grade 1 classroom was recruited to participate in the evaluation. The inclusion criteria for children’s participation were: (1) attendance of ≥80% over the school year at the time of recruitment, (2) no obvious disability, and (3) parental consent. All schools and all teachers agreed to participate in the study. One Grade 1 teacher from a control school left the school during the year and the children in that class were divided between the remaining Grade 1 classrooms. We were unable to get parent consent for one selected child and they were replaced by another randomly selected child.

Schools were randomised to intervention or control group in the summer preceding the intervention year. Randomisation was conducted by an independent statistician who was blind to the identity of the schools. All schools and teachers were recruited prior to randomisation. Children were recruited mid-way through the school year, after randomisation. All measurements were conducted after randomisation, as we collected data at post-test only. Ethical approval for the study was given by the School of Psychology Ethics and Research Committee, Bangor University on 14/01/2015, ref: 2014-14347 and the University of the West Indies Ethics Committee on 02/02/2015, ref: ECP 51, 2014/2015. Written informed consent was obtained from the primary school principal, all Grade 1 teachers and the parents of the children selected to participate in the evaluation. The study is registered in the International Standard Randomised Controlled Trial Number Register: ISCTRN 94883310.

### 2.2. Measurements

Outcome measurements included independent observations of teacher practices and classroom atmosphere, direct tests of children’s academic achievement using standardised tests, tester ratings of child self-regulation during the testing session, and teacher reports of child behaviour and their own wellbeing using interviewer-administered standardised questionnaires. Full details of all measures are given in Table 1. All measurements were taken by research assistants blind to the study design, hypothesis and group allocation.

#### 2.2.1. Primary Outcomes

The intervention aimed to prevent violence against children by teachers and to prevent the early development of antisocial behaviour, and so we had two primary outcome measures to allow us to evaluate the effect of the intervention on violence prevention at the level of the teacher and the level of the children (Table 1). Both primary outcomes were measured through observation and were based on measures we have used previously in Jamaican early childhood classrooms [17,18]. Observations are the gold standard for measuring behaviour and are less open to bias than teacher reports.

For teachers’ use of violence against children, event sampling over one full school day was used to record the number of times teachers used physical violence, verbal abuse or other violence. All behaviours were defined in a manual with examples and non-examples given under each category. The score represents the number of times the teacher used violence over the school day. A different observer visited the classroom on another day and measured the level of class-wide child aggression over six 20 min periods using a seven-point rating scale. The average score over the six periods was used in the analysis.

#### 2.2.2. Secondary Outcomes

Secondary outcomes included outcomes at the level of teacher/classroom and at the level of the individual child. The teacher/classroom measures included classroom atmosphere, a binary variable of teachers’ use of violence over two school days and teachers’ professional wellbeing. The individual child measures included child academic achievement, self-regulation, behavioural difficulties and prosocial skills.

Measures of classroom atmosphere included two observational measures: class-wide prosocial behaviour and quality of emotional support provided in the classroom (Table 1). The measure of class-wide prosocial behaviour was based on a rating scale used previously in Jamaica [17,18]. The quality of emotional support was measured using the subscales comprising the Emotional Support Domain in the Classroom Assessment Scoring System (CLASS-K-3) [21]. The CLASS instrument is widely used to measure the quality of early childhood classrooms and has been shown to predict children’s social and academic skills [22]. It has been shown to be valid in non-US contexts including in Chile and Ecuador [23,24]. These two measures of classroom atmosphere were conducted over six 20 min periods, concurrently with the measure of class-wide child aggression. The average score over the six observation periods were used in the analyses. During these six 20 min periods, observers also coded whether teachers used violence or not. Violence was scored in three categories (physical violence, verbal abuse or other violence), using the same definitions as for the primary outcome. These data were combined with data on teachers’ use of violence over one full school day to create binary variables of teachers’ use of violence, (including physical violence, verbal abuse, other violence and any violence) over two school days.

Teachers reported on their depressive symptoms, burn-out and teaching self-efficacy and on individual children’s behavioural difficulties and prosocial skills (Table 1). Children were tested individually using a battery of academic achievement tests and their behaviour during the testing session was rated by the tester (Table 1). All questionnaires and tests have been used previously with Jamaican teachers [20,25].

#### 2.2.3. Procedure for Data Collection and Quality Control

Seven research assistants (RAs) collected the outcome measurements. Two RAs conducted observations of teachers’ use of violence over one full school day; two RAs conducted observations of the classroom atmosphere and teachers’ use of violence over six 20 min periods during a different school day; two RAs conducted the individual child tests and one RA conducted the teacher interviews. Only one data collector was present in a classroom at a time. The research assistants were rotated across schools and collected data on equal numbers from each group.

Training for the research assistants conducting observations lasted four weeks: one week in office, two weeks of field training and one week of interobserver reliability testing in the field. For observational measures involving rating scales (class-wide child behaviour, level of emotional support), observers were trained until they reached a reliability ICC (intraclass correlation coefficient) >0.8 with the trainer. Interobserver reliabilities between the trainer and the observer were conducted on a minimum of 10% of all measures during data collection, with ICCs all >0.8. For the data collected through event sampling (teachers’ use of violence against children), interobserver reliabilities between the trainer and each observer during training and during data collection were ICC > 0.95, and this was maintained during data collection. Training for the research assistants conducting child tests and teacher interviews lasted 3 weeks: 1 week in office, 1 week field training and 1 week for inter-rater reliabilities. Inter-rater reliabilities between the research assistants and the trainer were >−0.95 on all measures during training and on 10% of measures during the study. All questionnaire measures had good internal consistency (Cronbach’s alpha: mean 0.90, range 0.85–0.94) and test–retest reliability over two weeks (ICC: mean 0.81, range 0.76–0.86). Test–retest (ICC) over two weeks for the child achievement tests were mean = 0.92, range = 0.75–0.99. Full details of the psychometric properties of each measure are shown in Supplementary Tables S1 and S2. All measures were collected at post-test only. It was not possible to collect baseline data because the intervention phase started at the beginning of the school year. For teachers: many schools rotate teachers so that the teachers teach at a different grade level each year, thus preventing us from collecting data in the previous school year. For children: Grade 1 is the first grade in primary school and so the children were newly enrolled at the school.

### 2.3. Sample Size

The study was conducted on a small scale as we were piloting the teacher-training intervention in a new context, rather than conducting a fully-powered efficacy trial of effectiveness. In a previous study in Jamaican preschools, a more intensive teacher-training intervention in classroom behaviour management led to very large effect sizes on teacher practices (mean ES = 2.2 SD) [18]. In the current study, for the primary outcomes of teachers’ use of violence and class-wide child aggression, with 27 teachers per group, a cluster size of 4 teachers per school and assuming an intracluster correlation of 0.15, we could detect a difference of 1 SD with 90% power and at a 0.1 level of significance. The remaining outcomes are secondary outcomes. For the secondary outcomes at the level of the teacher and classroom, we could detect a difference of 1 SD with 90% power at a significance level of 0.1, similar to the primary outcomes above. For individual child outcomes, with 108 children in each group, a cluster size of 16 children/school and assuming an intracluster correlation of 0.05, we could detect a difference of 0.5 SD with 85% power at 0.1 level of significance.

### 2.4. Teacher Reports on Use of Corporal Punishment

We conducted semi-structured individual interviews with a subsample of intervention teachers to ascertain their perspectives of the training programme and to further explore the effects of the programme on teachers’ use of violence against children. In this paper, we report the data from these interviews related to teachers’ use of violence only. A purposive sample of 18 teachers from the intervention group (2 to 3 teachers/school) were interviewed by a research assistant who had not been involved in delivering the intervention. Teachers were selected based on two criteria including dosage of intervention received (low, medium, high) and facilitator reports of teachers’ enthusiasm (low, medium, high). Teachers were asked to what extent the programme led to changes in their use of corporal punishment, the mechanism by which the training led to these changes (if relevant) and the extent to which teachers continued to use corporal punishment as a behaviour management strategy. The interviews were conducted at school at a time convenient for the teachers. Interviews were recorded and transcribed and key points around each theme were extracted. One teacher did not consent to be recorded and written notes were made during the interview.

### 2.5. The Intervention

The intervention involved training Grade 1 teachers in selected core content from the IRIE Classroom Toolbox [19]. The selected content focussed primarily on the use of positive and proactive strategies to promote children’s positive behaviour and prevent negative behaviour (e.g., use of praise, teaching classroom rules); content related to managing misbehaviour (e.g., withdraw attention, consequences, time-out) was not included. The reasons for including only the positive strategies were: (1) there was insufficient time to cover all of the content in the IRIE Classroom Toolbox and teachers need to be able to use the positive strategies before the negative strategies are introduced, (2) these were rated as effective, easy to use and the best liked components in previous studies with Jamaican preschool teachers [19], and (3) these strategies have been shown to be relatively easy for teachers to learn and are more acceptable to teachers [34]. Full details of the teacher-training intervention, using the relevant times from the TIDier checklist [35], are given in Box 1. Teachers from the control schools acted as a no treatment comparison group and they did not receive training sessions, in-class support, text messages or the intervention materials. However, all Grade 1 classrooms in participating intervention and control schools received additional educational resources including play dough, paints and paintbrushes, crayons, coloured pencils and drawing paper.

Box 1 **The Teacher-Training Intervention.**

**Content and Materials:** The intervention was based on selected core content from the IRIE Classroom Toolbox. The key concepts introduced were (1) teaching rules and routines, (2) using praise in the classroom and paying attention to positive behaviour, (3) being proactive to prevent child behaviour problems, (4) promoting children’s social–emotional competence, (5) interactive storybook reading and (6) promoting children’s active participation in teaching and learning activities.

Intervention materials for teachers include (1) a ‘Tools’ book which provides simple guidelines on how to use each strategy and the underpinning rationale, (2) an ‘Activity’ book of songs, games, activities and lesson plans, (3) three sets of picture cards to help teachers teach classroom rules, friendship skills and understanding emotions, (4) a ‘Problem-Solving Stories Book’ consisting of 14 pictorial stories depicting common classroom problems children encounter in school and strategies children can use to overcome them (e.g., how to share classroom materials).

 

**Procedures:** Teachers were invited to training sessions to introduce them to the strategies taught through the programme. The sessions included the use of demonstrations, practice activities with feedback and group discussions. Full details of the content and process for each training session were provided in a training manual.

Teachers’ implementation of the strategies introduced through the training sessions was supported through eight in-class support sessions. All sessions included three steps: (i) a brief planning discussion with the teacher (5 min), (ii) supporting the teacher in the classroom by modelling the strategies, prompting the teacher to use them, providing supportive feedback and helping the teacher to problem-solve (approximately 45 min) and (iii) debriefing and goal-setting (approximately 10 min). Teachers also received practical classroom assignments to be completed after each in-class support session and fortnightly text messages to remind and encourage them to use the strategies.

 

**Who provided:** Two female staff delivered the intervention. One senior facilitator delivered the workshop training sessions and a junior facilitator delivered the in-class support. The junior facilitator was supervised by the senior facilitator, who supported her in the classroom once a month and met fortnightly to discuss the progress made by each teacher. These two facilitators were trained and supported by the Principal Investigator (H.B.H.) and both facilitators joined weekly group supervision meetings with facilitators working on a similar intervention in Jamaican preschools.

 

**Where:** Teacher-training workshops were conducted primarily in a centrally located primary school in urban areas of Kingston, Jamaica.

 

**When, How and How Much:** Training workshops were held after school and/or in school holidays to fit around teachers’ schedules. Teachers were offered a total of 12 h of training workshop sessions. In-class support was provided to each intervention teacher once a month for 8 months (September to April) for approximately 1 hour each session.

 

**Fidelity:** The workshop facilitator completed a training protocol and self-evaluation after each training session; all of the prescribed content was covered. Nine teachers (33.3%) in the intervention group attended 9–12 h of training workshops, while 11 (40.7%) attended for at least 6 h. Two teachers (7.4%) attended none of the training sessions. Facilitators similarly covered all prescribed content in the in-class support sessions; 10 teachers (37%) participated in all eight in-class support sessions, 21 (78%) participated in six or more, 25 (93%) participated in four or more. The median number of hours training received (through workshops and in-class support) was 11 (range: 3–20, interquartile range: 7–16). Teachers were given classroom assignments after the first six in-class support sessions. Twenty-two teachers (81.5%) of teachers did at least one of these assignments, only eight teachers (30%) did three or more.

### 2.6. Statistical Analysis

All continuous outcome variables were checked for normality. Normality was rejected for teachers’ use of violence across one school day, teacher depression, teacher self-efficacy and three of the school achievement measures: word attack, maths reasoning and self-regulation. Teachers’ use of violence across one school day was normalised using a log transformation. A principal component factor analysis of the three measures of teacher wellbeing (depression, burnout and self-efficacy) produced one factor (Supplementary Table S3), and the factor scores were saved as regression scores in SPSS (v 23) (IBM Corporation, Armonk, NY, United States) and used in the analysis [36]. Factor analysis of the school achievement tasks, using Varimax rotation, produced two factors (Supplementary Table S4), and these scores were also saved as regression scores and used in the analyses. The effect of intervention on teacher, classroom and child outcomes was examined using multilevel multiple regression analyses to take into account the hierarchical nature of the data (children, nested in classrooms, nested in schools). For the analyses of the effect of intervention on the teacher and classroom measures (teachers’ use of violence against children over one school day, class-wide child aggression and prosocial behaviour, level of emotional support, and teacher wellbeing), observer/interviewer and intervention group were entered as fixed effects and school was entered as a random effect. In the analyses on individual child outcomes (child behaviour, self-regulation, and school achievement), child age and sex, tester/interviewer and intervention group were entered as fixed effects and school and teacher were entered as random effects. All multilevel analyses were conducted using MLWin (v. 2.10) (Centre for Multilevel Modelling, University of Bristol, Bristol, UK) [37].

## 3. Results

### 3.1. Sample

Sample characteristics are shown in Table 2. All teachers were female and all had completed a formal teacher training qualification. Teachers had been teaching for an average of fifteen years and there was a mean of thirty children in each class. There were no significant differences between the intervention and control groups on teacher and classroom characteristics. The mean age of the children selected to participate in the evaluation was seven years and there was a small, yet statistically significant, difference between the groups. Half of the children in the sample were boys.

### 3.2. Intervention Implementation

Teachers participated in a mean (SD) of 11.5 (5.1) hours of training, (including training sessions and in-class support sessions) out of a total of twenty hours (Panel 1). Teacher participation was affected by scheduling conflicts caused by other school commitments. This was especially relevant for the grade co-ordinators due to the administrative burden attached to their role. Teacher absence was another factor affecting participation: two teachers were often off school due to sickness and two teachers were often running errands during school time. Facilitators reported that the majority of teachers were willing to participate in the training programme and only two teachers were somewhat resistant.

### 3.3. Findings from the Impact Evaluation

Raw scores for all outcome measurements at post-test are shown in Table 3. For the primary outcomes, significant benefits of intervention were found for teachers’ use of violence against children (ES = −0.73, *p* < 0.0001); however, no significant benefits were found for class-wide child aggression (ES = −0.20, *p* = 0.47) (Table 4). Large significant benefits were found for the quality of the classroom environment in the emotional support domain (ES = 1.22, *p* < 0.0001), but there were no benefits to children’s class-wide prosocial behaviour and teacher wellbeing. For individual child measures, there were no benefits to child behavioural difficulties and prosocial skills by teacher report and no benefits to one factor of school achievement that reflected more formal test scores (e.g., spelling, phonics, maths calculation, reading). However, we did find benefits of 0.25 SD at the *p* < 0.1 level to the second factor of the school achievement tests that reflected the skills required for the process of learning (e.g., oral language, self-regulation).

Use of violence by teachers was common in Grade 1 Jamaican classrooms, with all teachers in the control group using violence at least once over two school days and only one teacher (<4%) not using physical violence during the period. There were significant differences in the prevalence of teachers’ observed use of violence over two school days between teachers in the intervention and control groups across all categories of violence (Table 5). However, only three intervention teachers (11.1%) used no violence over the two days of observation showing that although the intervention was successful at reducing violence against children, the majority of intervention teachers continued to use it as part of their behaviour management strategy.

### 3.4. Findings from Teacher Reports of Corporal Punishment

All 18 intervention teachers who participated in the semi-structured interview reported a reduction in their use of corporal punishment in the classroom as a result of the training. The most common reason given for this reduction was that they now had alternative strategies that worked to manage children’s behaviour (Table 6). Other reasons included: (1) the children were better behaved, (2) the teacher was better able to regulate her emotions, (3) using the strategies was less stressful than using corporal punishment, and (4) the teacher had better relationships with the children and viewed them more positively. Although teachers reported using less corporal punishment, all except one teacher reported that they continued to use corporal punishment at times. For some teachers, this was for specific child behaviours that they considered severe (especially antisocial behaviour such as hurting other children, spitting, stealing), whereas for others it was due to the strategies not working consistently or quickly enough. A significant minority of teachers also saw corporal punishment as an effective method for quickly dealing with problem behaviour, particularly as children were accustomed to this method of behaviour management in the home.

## 4. Discussion

Training Grade 1 primary school teachers in Jamaica in selected core content related to classroom behaviour management and promoting children’s social and emotional competence led to important benefits to teacher practices and child outcomes. These benefits included reductions in the frequency of teachers’ use of violence against children (ES = −0.73), increases in the emotional quality of the classroom environment (ES = 1.22) and benefits to children’s learning, specifically related to children’s oral language, self-regulation and reasoning skills (ES = 0.25). In addition, fewer teachers who participated in the intervention used violence against children (including physical violence, verbal abuse and other violence (e.g., intimidation)) over two full days of observation than teachers in the control group (11.1% vs. 0% for total violence respectively). These results from the impact evaluation were corroborated with the information from the in-depth interviews in which teachers reported using less corporal punishment after participating in the intervention programme. The intervention was relatively brief, with teachers receiving on average 11.5 h of training. Furthermore, teachers were only exposed to positive and proactive strategies of behaviour management (e.g., use of praise and positive attention, teaching and reinforcing classroom rules, promoting child interest and engagement in learning activities), with limited training on strategies to manage problem behaviour when it did occur, and no explicit focus on changing attitudes related to corporal punishment. It is promising that this short training programme led to improvements at multiple levels, including benefits to the classroom environment, teacher practices and individual child competencies. The intervention did not, however, lead to improvements in all measured outcomes. No benefits were found for observer ratings of class-wide child aggression and prosocial behaviour, teachers’ reports of their professional wellbeing, teacher-reported child behavioural difficulties and prosocial skills and direct testing of child academic achievement in basic reading, spelling and maths calculation skills.

The use of violence against children by teachers in Jamaican primary schools is extremely common, and the use of corporal and other harsh punishments are normative in Jamaica at school and at home [38,39]. The evidence that providing teachers with training in positive discipline methods leads to significant and meaningful reductions in teachers’ use of violence against children is important as it shows that a limited amount of training and support can lead to changes in teachers’ discipline strategies, even in a context in which the use of corporal punishment is widely used and accepted. The fact that the reductions in violence were accompanied by large increases in the quality of the emotional environment of the classroom indicates that teachers were using positive strategies to create a more supportive, nurturing and secure environment. These findings were corroborated through the in-depth individual interviews with intervention teachers, who reported using less harsh punishment with children because they had learned alternative positive strategies that worked to modify children’s behaviour, and helped to improve teacher-child relationships. This mechanism of reducing teachers’ use of violence by providing teachers with alternative effective discipline strategies is an emerging theme in the literature, and has been reported in studies with Jamaican preschool teachers [17,18,40] and Ugandan primary school teachers [41]. However, it is important to note that the majority of teachers continued to use violence at times, with only three (11.1%) intervention teachers using no violence over the two days of observation. Again, these results were supported by the in-depth interviews. The majority of teachers reported that they continued to use corporal punishment as a behaviour management strategy in some situations, especially for behaviours they considered to be particularly severe or when using the strategies did not lead to the desired outcome. Some teachers believed it is necessary to use corporal punishment, as this is what children are used to at home. In the evaluation of the Good School Toolkit in Ugandan primary schools, violence was reduced, but not eliminated [7], and teachers also reported similar beliefs around the use of corporal punishment at school [41]. These similarities across the studies are interesting, given that the interventions differed in many important ways. The Good School Toolkit (1) was implemented over 18 months, compared to 8 months in the current study, (2) included an explicit focus on changing teachers’ beliefs and use of corporal punishment, whereas in the current study, violence against children was not addressed directly but rather the focus was on training teachers in the use of new skills, and (3) involved the whole school rather than only teachers of a particular grade.

It is clear that teachers require additional support to further reduce the use of violence against children. A qualitative review of parenting interventions aimed at reducing child abuse found that teaching parental self-management strategies was an important component of effective programmes [42]. Explicit attention to promoting teachers’ self-regulatory capacities may help to further reduce teachers’ use of violence against children [43]. It is also important to include content on how to manage misbehaviour, including the more severe child behavioural difficulties, as teachers need alternatives to corporal punishment. The IRIE Classroom Toolbox includes a module on managing child misbehaviour and a module on behaviour planning. These modules were not covered in this study due to the short time available for training and the fact that learning the positive and proactive strategies are prerequisites for learning the negative techniques. Beliefs about the effectiveness and desirability of corporal punishment have been shown to predict its use [44], and hence addressing teachers’ beliefs and attitudes to corporal punishment may also be necessary. As corporal punishment is legal in Jamaican primary schools, enacting a legal ban in addition to training the teachers is also an important step towards its eradication. In this study, only teachers of Grade 1 children were trained, and it is important to combine this skills-based training with a whole-school approach to preventing violence against and among children [45]. The Ministry of Education is currently implementing the Positive Behavioural Intervention Support (PBIS) approach in primary schools across Jamaica. This involves guiding schools to develop whole school plans to address the school culture, along with behavioural supports at three levels of intensity, including universal (tier 1: for all children), selective (tier 2: targeted) and indicated (tier 3: needs-driven) supports to address the needs of all children within the school [46]. The teacher-training programme evaluated in this study can be implemented as a component of the support given for PBIS and has particular relevance at tiers one and two.

Based on previous qualitative evaluations of a teacher-training programme with Jamaican preschool teachers [19,40], we had also hypothesized that training teachers in classroom behaviour management could lead to higher levels of professional wellbeing among intervention teachers. However, no significant benefits were found in the impact evaluation, although in the in-depth interviews, some teachers did report that using positive strategies was less stressful than using harsh punishment and that they were calmer, less stressed and more relaxed after the training. Few studies have included measures of teacher wellbeing in evaluations of teacher-training interventions in LMIC and the results are mixed [47,48,49].

We had hypothesized that the changes in teacher practices, including using more positive and less negative strategies and a focus on teaching social and emotional skills to children, would lead to reductions in child aggression and increases in children’s prosocial behaviour. However, the intervention did not lead to benefits to the measures of class-wide or individual child behaviour. In previous studies in Jamaican preschools, a longer and more comprehensive teacher-training programme led to significant benefits to class-wide child behaviour [17,18]. Benefits of this more comprehensive training programme were also found for children at high risk for the early development of antisocial behaviour, including fewer conduct problems and increased social skills [16]. There are several potential reasons for the lack of benefits to child behaviour in this study: (1) the dosage of intervention may have been too low to lead to meaningful benefits to children’s behaviour, (2) training teachers in strategies to manage children’s misbehaviour (e.g., withdrawing attention, use of consequences, use of time-out, individual behaviour planning) may be essential core content and was omitted in this study, (3) the changes in children’s behaviour may emerge over time as they continue to be exposed to a more emotionally supportive classroom environment, (4) the effect sizes for class-wide child aggression and prosocial behaviour were −0.20 SD and 0.18 SD, respectively, and the study was not powered to detect differences of this magnitude, and 5) for the individual child outcomes, a random sample of children was evaluated rather than a high risk sample, and violence prevention interventions have been shown to benefit children at high risk the most [50]. Other studies involving training early childhood educators in LMIC have found significant benefits to teacher practices without accompanying benefits to child outcomes [51,52]. Large and sustained changes to the classroom environment may be required before child benefits become evident.

Perhaps surprisingly, we did find benefits to a measure of children’s early learning skills related to oral language and self-regulation skills (ES = 0.25), while no benefits were found to more formal measures of achievement, such as reading, spelling and calculation skills. Teachers were trained to map children’s actions with language, explicitly teach classroom rules, provide praise and encouragement to follow the rules, and to support children’s autonomy in the classroom. The benefits to children’s language and self-regulation skills may be a result of teachers’ use of these strategies. The intervention did not involve training in how to teach literacy and maths skills, and hence it is not surprising that no benefits were found on tests of these skills over the short time frame of the intervention. It is possible that benefits may emerge over time, or it may be that a more explicit focus on early academic skills is required to benefit child achievement.

The training programme was delivered through a combination of workshop sessions and in-class support. These are common methods for in-service teacher-training initiatives globally [53]. We faced several challenges in implementing the programme through in-service training initiatives. Attendance at workshop training sessions was hindered by the fact that the programme was conducted as a research programme and was not part of the professional development opportunities required by the Ministry of Education (MOE). Hence, when teachers were organizing their professional development activities, the MOE training initiatives took precedence. Integrating the training programme into MOE in-service training would ensure that all activities were scheduled into the school calendar at the beginning of the school year, thus maximizing the likelihood that teachers would attend the training sessions. Scheduling in-class support sessions was challenging due to the competing demands of other school commitments, especially for teachers with additional responsibilities such as the grade co-ordinator. The training programme is also suitable for integration into pre-service training initiatives, and could be incorporated into the teaching practice component of the teacher-training course to ensure trainee teachers receive support in the classroom in addition to receiving the workshop training sessions.

The study has several strengths. Schools were randomized to intervention group, all schools and teachers were also recruited prior to randomization and attrition was low, with only one teacher lost between randomization and post-test measurements. There were no differences between the groups on teacher and classroom characteristics, suggesting that the randomization led to balanced groups. This means that we can be reasonably confident that the differences reported were due to the additional training the intervention teachers received. The primary outcomes involved independent observations of teachers’ and children’s behaviours by observers who were masked to the study design, hypothesis and group allocation. Observations are less open to bias than teacher report. All questionnaire and child test measures had good psychometric properties, had been used previously in Jamaica and were administered by masked data collectors. For individual child measures, the children were randomly selected to participate in the evaluation. There was a small, yet significant difference in child age across the study groups; child age was controlled in all regressions on individual child outcomes. The study also had several limitations. The sample size was small, and hence we did not have power to detect small differences between the groups. We were not able to collect baseline data, and hence we were unable to control for initial scores and we do not know if the groups were equivalent at baseline on all outcome measures. Teachers were aware of their intervention status and they may have behaved differently during the observation period. However, observations were conducted over two full days, and there is evidence that conducting observations over a whole day minimizes reactivity to being observed [54]. Children were excluded from the individual assessments if they had school attendance lower than 80%; it is likely that low school attendance would reduce the benefits of the intervention.

## 5. Conclusions

Training Jamaican primary school teachers in the use of positive discipline strategies led to reductions in the frequency of teachers’ use of violence against children and large benefits to the quality of the classroom environment. Benefits were also found to the prevalence of teachers’ use of violence, with fewer intervention teachers using physical, verbal or other violence over two full days of observation. We also found benefits to a measure of children’s early learning skills relating to oral language, reasoning and self-regulation. The fact that a relatively low dose of intervention led to these benefits is encouraging, and the programme has the potential to be integrated into both in-service and pre-service teacher training. No benefits were found to class-wide or individual child behaviour, and a more comprehensive programme may be required for child behaviour benefits. Further research is required to examine how this teacher-training programme can be effectively integrated into the training provided through the Ministry of Education in a sustainable way. The training programme is suitable for teachers in Grades 1–3 (catering to children aged up to 8 years), and as only Grade 1 teachers were trained in this study, research is also needed to evaluate its effectiveness with teachers in Grades 2 and 3.

## Figures and Tables

**Table 1:** Outcome measures.

	Description of Measures Used
**Teachers Use of Violence Against Children ^1^**	**Continuous observations of teacher behaviour over one school day using event sampling. Total score is the sum of teachers’ use of violence in each category.**
Physical punishment	Hitting with hand, hitting with object, forcefully pushing or pulling, shaking, pinching, poking, throwing an object at the child, making the child stand or kneel in uncomfortable positions (e.g., stand with hands out to the side).
Verbal abuse	Calling the child by a derogatory name (e.g., idiot, dummy, fool), threatening physical punishment, threatening the child in way that would frighten them (e.g., threaten to lock them up), threatening to withhold food, rejection, encouraging other children to harm, insult or exclude the child (e.g., encouraging a child to hit another child).
Other abuse	Intimidation (e.g., banging a stick hard on the desk in front of a child), non-verbal threat (e.g., using stick/ruler to threaten child with physical punishment).
**Observations of the Classroom Environment**	**Classroom observations over six 20 min periods on a seven point rating scale (1–7) over one school day: mean score over six observations used in analyses.**
Class-wide child aggression ^1^	The score for class-wide aggression reflects the frequency, intensity and number of children involved in aggressive acts. Higher scores indicate more aggression.
Class-wide child prosocial behaviour	The score for class-wide prosocial behaviour reflects the frequency, intensity and number of children involved in prosocial acts (i.e., sharing, helping and cooperating). Higher scores indicate more prosocial behaviour.
Levels of emotional support	Emotional support was measured using the Classroom Assessment Scoring System (CLASS)- K-3 [21]. The score is a composite of four rating scales: Positive Climate, Negative Climate, Teacher Sensitivity, and Regard for Student Perspectives. Higher scores indicate a more emotionally supportive environment.
**Teachers’ Use of Violence (Binary)**	**Observations of teacher behaviour over two full days (binary variable).**
Teachers’ use of violence	Event sampling of teachers’ use of violence against children (physical punishment, verbal abuse, other abuse) over one full school day and over five 20 min observation periods on another school day. The scores represent whether or not the teacher used physical violence, verbal abuse, other violence and violence of any type against children over the two days of observation.
**Child behaviour**	**Assessed through teacher report. Total score for each scale used in the analyses.**
Behavioural difficulties and prosocial skills	Strengths and Difficulties Questionnaire by teacher report: a total difficulties (20 questions) and prosocial (5 questions) score were computed [26].
**School Achievement**	**Assessed through direct testing and tester ratings. Factor scores used in analyses.**
Oral language skills	Understanding Directions and Story Recall from the Woodcock–Johnson III Tests of Achievement [27].
Reading	Letter word identification and Passage Comprehension from the Woodcock–Johnson (WJ) III Diagnostic Reading Battery [28].
Phonics	Word attack and spelling of sounds subscales from the Woodcock–Johnson III Diagnostic Reading Battery [28].
Spelling	Spelling subscale from the Woodcock–Johnson III Tests of Achievement [27].
Maths	Calculation and Reasoning and Concepts subscales from the Woodcock–McGrew–Werder Mini-Battery of Achievement [29].
Self-regulation	Rated during the test session using eleven four point scales from the Preschool Self-Regulation Assessment: Assessor Report [30]. Items related to child attention and child impulse control. Higher scores indicate higher self-regulation.
**Teacher Wellbeing**	**Assessed through teacher report. Factor score using in the analysis.**
Depressive symptoms	Frequency of depressive symptoms using the Centre for Epidemiological Studies Depression Scale [31]. The scale consists of 20 questions scored on a four point scale (0–3). Higher scores indicate higher levels of depressive symptoms.
Burnout	Teacher burnout measured using the Teacher Burnout Scale [32]. The scale consists of 20 questions scored on a five point scale (1–5).
Teaching self-efficacy	Four subscales from the Bandura’s Teaching Self-Efficacy Scale: instructional, disciplinary, enlisting parent involvement and creating a positive school climate [33]. The scale consists of 25 questions scored on a seven point scale (1–7).

**^1^** Primary outcomes.

**Table 2:** Teacher, classroom and child characteristics by intervention group.

	Intervention	Control	*p*-Value
Teacher and Classroom Characteristics	*n* = 27	*n* = 27	
% female	100%	100%	1.00
% completed high school	100%	100%	1.00
% with a Dip.Ed or B.Ed	100%	100%	1.00
Teacher age ^1^	39.5 (10.9)	43.2 (10.2)	0.23
Number of years teaching ^1^	14.9 (9.9)	16.8 (9.8)	0.50
Number years teaching at this school ^1^	11.2 (9.7)	10.0 (8.1)	0.63
No. children in class ^1^	30.2 (6.5)	30.0 (5.2)	0.91
Child Characteristics	*n* = 108	*n* = 112	
Child age ^1^	7.00 (0.36)	6.90 (0.30)	0.01
% male	54 (50.5%)	53 (49.5%)	0.69

^1^ Values are mean (SD).

**Table 3:** Classroom, teacher and child outcomes at post-test by intervention group ^1^.

**Teacher and Classroom Outcomes**	**Intervention *n* = 27**	**Control *n* = 27**
Primary Outcomes		
Teachers’ use of violence over one school day (median, range)	4 (0–70)	12 (0–81)
Children’s class-wide aggression ^2^	4.16 (1.25)	4.40 (1.20)
Secondary Outcomes		
Emotional quality of classroom ^2^	4.10 (0.66)	3.48 (0.51)
Children’s class-wide prosocial behaviour ^2^	2.33 (0.73)	2.21 (0.67)
Teacher depression ^3^ (median, range)	9.0 (0–46)	8.0 (0–48)
Teacher burnout ^4^	33.8 (11.8)	34.2 (12.2)
Teacher self-efficacy ^5^ (median, range)	142 (72–172)	127 (102–164)
**Individual Child Outcomes**	***n* = 108**	***n* = 112**
Strengths and Difficulties Questionnaire		
Behavioural difficulties ^6^	9.12 (6.50)	9.55 (5.94)
Prosocial behaviour ^7^	6.82 (2.60)	7.20 (2.36)
Child Academic Achievement		
Understanding directions	25.75 (6.51)	24.00 (7.06)
Story recall	30.22 (15.07)	30.38 (18.28)
Letter word ID	27.54 (9.09)	28.00 (9.60)
Reading comprehension	13.63 (6.30)	14.01 (6.54)
Word attack, median (range)	6 (2–31)	6 (1–31)
Spelling of sounds	20.41 (7.66)	19.33 (7.58)
Spelling	21.22 (5.66)	21.88 (5.83)
Calculation	7.49 (2.89)	6.79 (3.16)
Maths reasoning (median, range)	28 (18–35)	27 (10–32)
Self-regulation ^8^ (median, range)	31 (13–33)	31 (15–33)

^1^ All values are mean (SD) unless otherwise specified; ^2^ Rated on a seven point scale (1–2 = low, 3–5 = medium, 5–7 = high); ^3^ Depressive symptoms: min = 0, max = 60; ^4^ Burn out: min = 20, max = 100; ^5^ Teaching self-efficacy: min = 25, max = 150; ^6^ Behavioural difficulties: min = 0, max = 40; ^7^ Prosocial behaviour: min = 0, max = 10; ^8^ Self-regulation: min = 0, max = 33.

**Table 4:** Effect of intervention on teacher, classroom and child outcomes ^1,2^.

Measure	Regression Coefficient B (95% CI)	ICC ^3^	Effect Size ^4^ (95% CI)	*p*-Value
Primary Outcomes				
Teachers’ use of violence against children ^5^	−0.38 (−0.68, −0.08)	0.05	−0.73 (−0.15, −1.31)	<0.0001
Class-wide child aggression ^6^	−0.24 (−0.88, 0.40)	0.00	−0.20 (−0.73, 0.33)	0.47
Secondary Outcomes: teacher and classroom outcomes				
CLASS: emotional support ^6^	0.62 (0.29, 0.95)	0.09	1.22 (0.57, 1.87)	<0.0001
Class-wide child prosocial behaviour ^6^	0.12 (−0.24, 0.48)	0.00	0.18 (−0.36, 0.72)	0.52
Poor teacher wellbeing ^7^	−0.11 (−0.63, 0.42)	0.00	−0.11 (−0.63, 0.43)	0.69
Secondary Outcomes: individual child outcomes				
Child behavioural difficulties ^8^	−0.64 (−3.17, 1.89)	0.06	−0.11 (−0.53, 0.32)	0.62
Child prosocial behaviour ^8^	−0.32 (−1.27, 0.63)	0.03	−0.14 (−0.54, 0.27)	0.50
Academic achievement factor ^9^	−0.01 (−0.55, 0.55)	0.21	−0.004 (−0.54, 0.54)	0·99
Language & self-regulation factor ^10^	0.25 (−0.02, 0.52)	0.00	0.25 (−0.02, 0.52)	0·07

^1^ Analysis adjusting for observer/interviewer as fixed effects and school and classroom as random effects. ^2^ Intervention group = 1, control group = 0. ^3^ Intra-cluster correlation coefficient; ^4^ The effect size is the regression coefficient divided by standard deviation of control group at post-test. ^5^ Log transformed score; observations taken over one full school day; ^6^ Observer ratings over six 20 min periods over 1 school day; ^7^ Factor score of frequency of depressive symptoms, teacher burn out & self-efficacy; ^8^ Teacher reported child behaviour using the Strengths and Difficulties Questionnaire; ^9^ Factor score: reading, spelling, phonics, maths calculation (see Supplementary Table S4). ^10^ Factor score: oral language, self-regulation, maths reasoning (see Supplementary Table S4).

**Table 5:** Teachers’ use of violence over 2 days at post-test by intervention group.

	Intervention *n* = 27	Control *n* = 27	*p*-Value
No physical violence	6 (22.2%)	1 (3.7%)	0.04
No verbal abuse	10 (37%)	3 (11.1%)	0.03
No other violence	22 (81.5%)	13 (48.1%)	0.01
No violence of any type	3 (11.1%)	0 (0%)	0.08

**Table 6:** Results from Semi-Structured Interviews.

Subtheme	Examples of Quotes
**Reasons for using less corporal punishment (CP)**	
The strategies work, so do not need to use CP (14)	“It lessen, put it this way, what I would normally slap them for, I don’t slap them for that anymore—because I have the strategies that helps me.” “I can’t tell when last I have to use the strap. I don’t need to use it. It has worked tremendously. The program make me not use it, all the skills, all of them work: the rules, the praise, strategic and labelled praise and friendship skills too.”
Children are better behaved (5)	“They are behaving in the way you want them to behave so you don’t have to reprimand them because they are actually doing what is expected of them.” “The more I use the strategies the more the appropriate behaviour is displayed. So that in itself, allowed me to use less.”
Teacher better able to stay calm (7)	“It helps me to be more relaxed than I used to be. I used to get easily upset but it has calmed me, helped me to relax and basically to be more understanding.”
Using the strategies is less stressful than using CP (5)	“Less strenuous on teacher. The energy you would use to slap them, you use the strategies. Saves you physically and mentally, voice don’t go and you don’t have to be hitting children.” “You feel more comfortable and more relaxed. So instead of doing corporal punishment—that really takes away from your peace and your sanity.”
Better relationship with children (5)	“It makes me see the good. I see children who are kind and helpful. So, it allows you to see them in another light. When you praise them—you see the good in them”
**Reasons for continuing to use corporal punishment (CP)**	
It works (5)	“Everybody pays attention, yes and they stop what they’re not supposed to do ’cause they’re afraid of the stick.”
CP is used at home (5)	“Because it’s what they are used to at home, once they see the strap then they will keep quiet.”
Use when other strategies don’t work (5)	“You know sometimes you do some of the strategies and the children don’t respond to it and you tend to want to get the strap.”
For antisocial behaviours (8)	“I don’t slap often but I do slap if a child is behaving bad, like spitting on a next child. Or if they do something bad to another child like slap them. I don’t normally do it—they have to do something bad and out the norm.” “I still beat but not often now. For stealing, that was the last thing I beat for.”

Numbers in parentheses indicate the number of teachers who mentioned the subtheme.

## Supplementary Materials

The following are available online at https://www.mdpi.com/1660-4601/16/15/2797/s1, Table S1: Internal reliability and test–retest of questionnaire data; Table S2: Test–retest of child school achievement tests; Table S3: Factor analyses the measures of teacher wellbeing at post-test; Table S4: Factor analysis of the child school achievement tests at post-test.
