# Supplementary material for: Evaluation of a Violence-Prevention Programme with Jamaican Primary School Teachers: A Cluster Randomised Trial
Source: Int J Environ Res Public Health. 2019 Aug 6;16(15):2797. doi: 10.3390/ijerph16152797 (PMC6696405; doi:10.3390/ijerph16152797)
Supplement: Supplementary file 1 [file ijerph-16-02797-s001.pdf]

Supplementary Tables

Table 1. Internal reliability and test-retest of questionnaire data.

	Cronbach's alpha	Test-retest [1] (ICC [2])
Teacher wellbeing		
Teacher depressive symptoms	0.94	0.76
Teacher burn-out	0.92	0.86
Teacher self-efficacy	0.94	0.81
Child behaviour by teacher report		
SDQ child behaviour difficulties	0.85	0.80
SDQ child prosocial behaviour	0.86	0.80

[1] Over 2 weeks: n=20 for all measures; [2] ICC: intraclass correlation coefficient.

Table 2. Test-retest of child school achievement tests.

	Test-retest [1] (ICC [2])
Following directions	0.88
Story recall	0.75
Letter-word identification	0.98
Reading comprehension	0.98
Word attack	0.94
Spelling of sounds	0.98
Spelling	0.94
Maths calculation	0.99
Mathematical reasoning	0.95
Self-regulation	0.85

[1] Over 2 weeks: n=20 for all measures; [2] ICC: intraclass correlation coefficient.

Table 3. Factor analysis the measures of teacher well-being at post-test.

	Factor 1
Teacher burn-out	0.89
Teacher depressive symptoms	0.80
Teaching self-efficacy	−0.73
Variance explained = 65.55%	

Table 4. Factor analysis of the school achievement tests at post-test.

	Factor 1	Factor 2
Letter word ID	0.94	
Spelling	0.92	
Reading comprehension	0.85	
Word attack	0.85	
Spelling of sounds	0.82	
Calculation	0.66	
Maths reasoning	0.58	0.54
Story recall	0.44	0.44
Self-regulation		0.78
Understanding directions		0.65
Variance explained = 67.75%		
